# Acute hepatitis with autoimmune features after COVID-19 vaccine: coincidence or vaccine-induced phenomenon?

**DOI:** 10.1093/gastro/goac014

**Published:** 2022-04-27

**Authors:** José M Pinazo-Bandera, Alicia Hernández-Albújar, Ana Isabel García-Salguero, Isabel Arranz-Salas, Raúl J Andrade, Mercedes Robles-Díaz

**Affiliations:** 1 Division of Gastroenterology and Hepatology, Instituto de Investigación Biomédica de Málaga-IBIMA, Hospital Universitario Virgen de la Victoria, Universidad de Málaga, Málaga, Spain; 2 Division of Anatomical Pathology, Hospital Universitario Virgen de la Victoria, Málaga, Spain; 3 Centro de Investigación Biomédica en Red de Enfermedades Hepáticas y Digestivas (CIBERehd), Madrid, Spain

## Introduction

Autoimmune diseases result from a breach of immunological self-tolerance and tissue damage by autoreactive T lymphocytes. Severe acute respiratory syndrome coronavirus-2 (SARS-CoV-2) infection is characterized by an inflammatory dysregulation that has been associated with the development of autoimmune processes [1].

Molecular mimicry has been suggested as a potential mechanism for these associations as well as ‘bystander activation’ where the infection may lead to activation of antigen presenting cells that may activate autoreactive T-cells, with the production of pro-inflammatory mediators and tissue damage [1].

There is a potential antigenic cross-reactivity between SARS-CoV-2 and human tissue possibly linked to an increase in autoimmune diseases. A recent study showed that antibodies against the spike protein S1 of SARS-CoV-2 had high affinity against some human tissue proteins such as transglutaminase 2 and 3, or myelin basic protein, among others [2].

As both mRNA vaccine (Comirnaty BioNTech BNT162b2 and Spikevax ARNm-1273) and vectorial vaccine (ChAdOx1nCoV-19 Vaxzevria/Covishield) give rise to the production of protein S, the antibodies produced against this protein after vaccination may also trigger autoimmune conditions in predisposed individuals.

Thirteen case reports (including 16 patients) have recently reported an association between COVID-19 vaccines and acute hepatitis development [3–15].

Here we report two new cases of liver injury possibly related to COVID-19 vaccination.

## Case 1

A 77-year-old woman developed intense malaise, vomiting and disorientation 2 days after receiving the second dose of Comirnaty vaccine and was hospitalized the following day. She did not have a history of autoimmune disorders. She denied alcohol drinking and was on long-term therapy with bromazepam, losartan, and omeprazole. Her previous liver tests back in 2020 were normal.

Physical examination was normal except for scleral icterus. Liver test showed acute hepatocellular injury: total bilirubin (TB) 3.1 mg/dL (reference, <1 mg/dL), aspartate aminotransferase (AST) 474 U/L (reference, <40 UI/L), alanine aminotransferase (ALT) 552 U/L (reference, <40 U/L), and alkaline phosphatase (ALP) 159 U/L (reference, <117 U/L). Immunoglobulin G levels were within normal ranges (reference, 800–1,600 mg/dL), while anti-nuclear antibody and anti-mitochondrial antibody M2 were detected with 1/160 and 1/40 titre, respectively. Human leukocyte antigen (HLA) testing was positive for HLA-DR4. All the other possible aetiologies were ruled out.

The patient was discharged and closely monitored. Due to increased transaminase levels, she underwent a liver biopsy (Supplementary Figure 1.1), which showed findings compatible with autoimmune hepatitis (AIH).

Prednisone 60 mg/day on tapering dose was initiated and 3 weeks later liver test had markedly improved. Azathioprine was added 2 months later, but it had to be withdrawn due to rash. Prednisone was then replaced by budesonide 9 mg/day. Five months after onset, transaminases were within the normal range; however, the subject was hospitalized with neurologic symptoms in relation to brain lesions in both hemispheres of probable infectious origin and died 1 month later.

## Case 2

A 23-year-old man presented with mononucleosis syndrome-like symptoms and jaundice at the emergency room, 10 days after receiving the second dose of Spikevax vaccine. He did not suffer from previous autoimmune disorders. He denied having taken any conventional drug treatments as well as alcohol consumption.

Physical examination was unremarkable except for scleral icterus. Liver tests showed acute hepatocellular injury: TB 2.3 mg/dL, AST 702 U/L, ALT 587 U/L, and ALP 202 U/L. Immunoglobulin G levels were minimally elevated (1,647 mg/dL), while autoantibodies resulted as negative. HLA testing was positive for HLA-DR3. Serology ruled out viral causes and abdominal ultrasonography was normal. After admission to the hospital, a thoracic-abdominal scan was performed and revealed generalized lymphadenopathy.

He underwent a liver biopsy (Supplementary Figure 1.2), which showed findings compatible with AIH.

Prednisone 60 mg/day on tapering dose was initiated and 1 month later lymphadenopathies were undetectable and liver test had significantly improved. Three months after onset, transaminases were within the normal range and he is still on low-dose prednisone 10 mg/day.

## Discussion

These new cases of liver injury compatible with AIH, which developed post COVID-19 vaccination, along with 13 prior published case reports (16 patients) reinforce that this association could be more than coincidental. In the previously published case reports, all the patients, except three, were females and their age ranged from 35 to 80 years [3–15]. Twelve of these patients received one of the mRNA vaccines [3, 5–12, 14, 15], while four patients received vectorial vaccines [4, 12, 13]. In 6 of the 16 patients, liver biopsy revealed infiltration with eosinophils [3, 4, 7, 9, 14] and IgG levels were increased in 12 cases [4–12, 15].

Fourteen reported patients were successfully treated with prednisolone whereas two died due to acute liver failure [4, 12] (Table 1).

**Table 1.** Characteristics of patients with liver injury after SARS-CoV-2 vaccine (published cases and two new cases)

Author	Vaccine	Dose	Days until clinical onset	Gender	Age	Liver-injury pattern	Autoimmune disease history	Auto- antibodies	IgG	Biopsy		Steroid response	Death
										Compatible (Yes/No)	Eosinophils infiltration (Yes/No)		
**Bril *et al.*** [3]	Comirnaty BioNTech BNT162b2	1st	13	F	35	Hep	None	ANA Anti-dsDNA	Normal	Yes	Yes	Yes	No
**Rela *et al.*** [4] **(2 cases)**	ChAdOx1nCoV-19 Covishield (both patients)	NA	20	F	38	NA^a^	None	ANA	High	Yes	Yes	Yes	No
		NA	16	M	65	NA^a^	None	NA	NA	Yes	Yes	No	Yes
**Rocco *et al.*** [5]	Comirnaty BioNTech BNT162b2	2nd	7	F	80	Hep	Hashimoto disease	ANA	High	Yes	No	Yes	No
**Londoño *et al.*** [6]	Spikevax, ARNm-1273	2nd	7	F	41	Hep	None	ANA SMA SLA LC-1	High	Yes	No	Yes	No
**Tan *et al.*** [7]	Spikevax, ARNm-1273	1st	35	F	56	Hep	None	ANA SMA	High	Yes	Yes	Yes	No
**McShane *et al.*** [8]	Spikevax, ARNm-1273	1st	4	F	71	Hep	None	SMA	High	Yes	No	Yes	No
**Ghielmet-ti *et al.*** [9]	Spikevax, ARNm-1273	1st	7	M	63	Hep	None	ASMA ANCA ANA	High	Yes	Yes	Yes	No
**Garrido *et al.*** [10]	Spikevax, ARNm-1273	1st	14	F	65	Hep	None	ANA	High	Yes	No	Yes	No
**Avci *et al.*** [11]	Comirnaty BioNTech BNT162b2	NA	14	F	61	Mix	Hashimoto disease	ANA SMA	High	Yes	No	Yes	No
**Erard *et al.*** [12] **(3 cases)**	Spikevax, ARNm-1273 (two first patients) ChAdOx1nCoV-19 Vaxzevria (third one)	2nd	10	F	80	NA^a^	None	Negative	High	Yes	No	Yes	No
		1st	21	F	73	NA^a^	None	Negative	High	Yes	No	Yes	No
		1st	20	F	68	NA^a^	None	Negative	High	Yes	No	No	Yes
**Clayton-Chubb *et al.*** [13]	ChAdOx1nCoV-19 Vaxzevria	1st	26	M	36	Hep	None	ANA	Normal	Yes	No	Yes	No
**Lodato *et al.*** [14]	Comirnaty BioNTech BNT162b2	1st	15	F	43	NA^a^	None	Negative	Normal	Yes	Yes	Yes	No
**Vuille-Lessard *et al.*** [15]	Spikevax, ARNm-1273	1st	3	F	76	Hep	Hashimoto disease	ANA	High	Yes	No	Yes	No
**Pinazo *et al.* (2 cases)**	Comirnaty BioNTech BNT162b2 (First one) Spikevax, ARNm-1273 (second one)	2nd	2	F	77	Hep	None	ANA AMA Negative	Normal	Yes	Yes	Yes	Yes^b^
		2nd	10	M	23	Hep	None		High	Yes	No	Yes	No

M, male; F, female; NA, not available; Hep, hepatocellular; Mix, mixed; IgG, immunoglobulin G; ANA, anti-nuclear antikor; SMA, smooth muscle antibodies; dsDNA, double-stranded DNA antibodies; LC1, liver sitozol antibody; anti-SLA, soluble liver antigen antibodies; ANCA, anti-neutrophil cytoplasmic antibodies; AMA, anti-mitochondrial antibodies.

a ALP (alkaline phosphatase) not available.

b The patient died due to an extrahepatic cause (brain lesions in both hemispheres of probable infectious origin).

In both cases of the present study, a number of laboratory (including HLA testing) and histological features supported the autoimmune nature of the liver injury. In our first case, the short period elapsed after vaccine administration, the laboratory and histopathological findings (showing moderate liver fibrosis), the positive HLA-DR4, and the response to therapy suggest unmasking of AIH by the vaccine. However, in our second case, the medical history negative for liver and autoimmune diseases, the short time interval after vaccination, the typical onset of symptoms to which was added generalized lymphadenopathy, the elevated immunoglobulin G levels, the positive HLA-DR3, histopathological findings with absence of liver fibrosis, and the response to therapy reinforce the hypothesis of SARS-CoV-2 vaccine as a trigger of an autoimmune liver injury debut. We realize that there are no pathognomonic (laboratory or histological) features of AIH, but the appropriate exclusion of viral and metabolic causes of liver injury makes the autoimmune mechanisms the more likely explanation for both cases.

Taking into account the large number of vaccinated subjects worldwide, the suspicion of vaccine-related AIH carries important clinical implications. It is unknown whether prolonged immunosuppression would be required in these cases or whether re-exposure to a new dose of COVID-19 vaccine might trigger fulminant liver injury. Nevertheless, the risk of receiving another dose must be balanced against the risk of contracting SARS-CoV-2 infection. In addition, it remains unclear whether patients who have developed liver injury after vaccination with one type of vaccine can receive other COVID-19 vaccine with a different mechanism of action.

Post COVID-19 vaccination, AIH has been rarely reported so far [3–15], which might be due to either minimal awareness of this disease or because patients without jaundice often do not seek medical attention. However, given the growing number of cases compatible with AIH reported after SARS-CoV-2 vaccination, regulators should consider the inclusion of this potential adverse event in the label of COVID-19 vaccines.

In conclusion, clinicians should be aware of the potential association between the vaccines and the onset of immune mediated disorders such as AIH. However, this rare complication should not discourage people from getting vaccinated.

## Supplementary Data

Supplementary data is available at *Gastroenterology Report* online.

## Authors’ Contributions

J.M.P.B., A.H.A., and M.R.D.: Patient care, writing of the manuscript, and revision of the final version of the manuscript. A.I.G.S. and I.A.S.: Pathology reading and revision of the final version of the manuscript. R.J.A.: writing of the manuscript and revision of the final version of the manuscript. All authors read and approved the final manuscript.

## Funding

Instituto de Investigación Biomédica de Málaga-IBIMA, Hospital Universitario Virgen de la Victoria, Universidad de Málaga, Málaga, Spain, Centro de Investigación Biomédica en Red de Enfermedades Hepáticas y Digestivas (CIBERehd), Madrid, Spain. Grants of Instituto de Salud Carlos III cofounded by Fondo Europeo de Desarrollo Regional—FEDER [contract numbers: PI18/00901, UMA18-FEDERJA-193], COST ACTION CA17112-Prospective European Drug-Induced Liver Injury Network and IMI2- Translational Safety Biomarker Pipeline (TransBioLine Horizonte 2020). CM21/00074 (Rio Hortega contract: J.M.P.B.).

## Conflict of Interest

None declared.

## Supplementary Material

goac014_Supplementary_Data
