# Supplementary figures and images for: Acute hepatitis with autoimmune features after COVID-19 vaccine: coincidence or vaccine-induced phenomenon?
Source: Gastroenterol Rep (Oxf). 2022 Apr 27;10:goac014. doi: 10.1093/gastro/goac014 (PMC9046091; doi:10.1093/gastro/goac014)

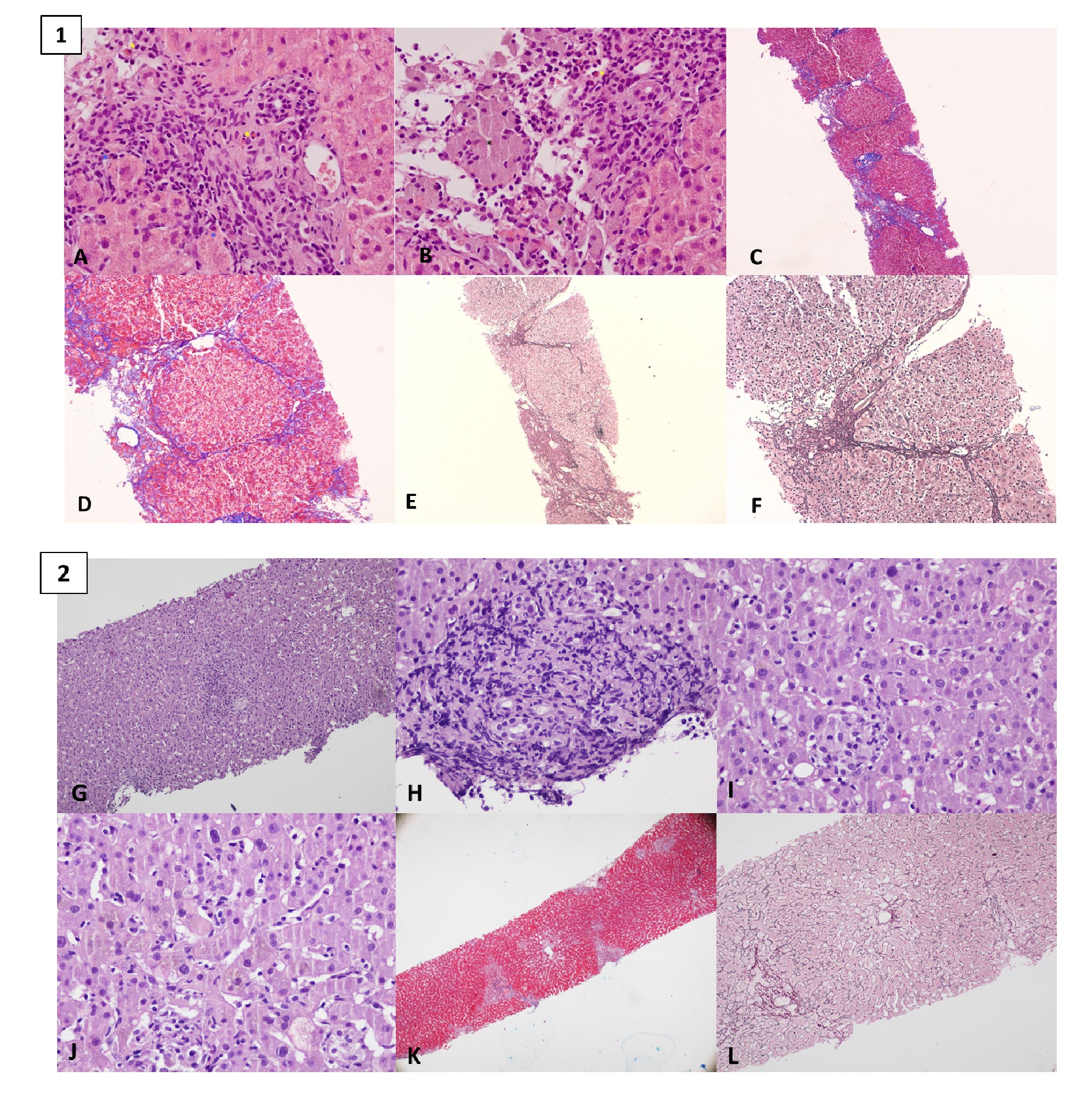

Supplement: goac014_Supplementary_Data [file goac014_supplementary_data.jpeg]
